# PRESERVING CELL HOMEOSTASIS AS AN AGING MODULTATION STRATEGY

**DOI:** 10.1093/geroni/igae098.2938

**Published:** 2024-12-31

**Authors:** Dennis Lox

**Affiliations:** Sports and Regenerative Medicine, Clearwater, Florida, United States

## Abstract

The cell desires to maintain homeostasis. The most efficient and productive method to achieve cell homeostasis, is utilization of the Longevity Pathway AMPK-SIRT1. This allows processes to repair, induce positive patterns that lead to systemic efficiency. The Longevity Pathway may be activated by exercise, drugs, MOTS-c (a mitochondrial derived peptide), Irisin (a adipomyokine Sestrin), and circadian clock genes. Some activators display synergistic effects with others and exercise. Upon activation of AMPK-SIRT1, molecular and cellular functions occur, allowing nuclear transfer of P53 and FOXO’s, this prevents cytoplasmi binding of P53-FOXO4, avoiding the deleterious effects of overactive mTOR, cell senescence and the SASP, upregulation of NF-kB, production of inflammatory cytokines, DAMP, and HMGB. This sets up the continuous feedback loop of continuing the cycle, and interactions with the of Hallmarks of Aging, compounding the process of driving further aging and disease development. Homeostasis occurs when P53 and the FOXO’s are translocated to the nucleus, allowing DNA repair, mitochondrial biogenesis, NRF1,2, and PPAR transformation, production of more NAD+ occurs, which is needed in the pathway to function, and lastly increased exercise capacity. As the GI Biome is now a Hallmark of Aging, Starting with a proper diet and exercise to initiate the Longevity Pathway to allow cell homeostasis, increasing cell efficiency, and decreasing deleterious pathways. Further targets for personalized treatment may better address human diversity.

